# Chondroid Soft Tissue Tumors With FOS::PABPN1 Fusion: A New Entity? About Two Cases

**DOI:** 10.1002/gcc.70157

**Published:** 2026-08-07

**Authors:** Jinane Kharmoum, Alexendra Meurgey, Daniel pissaloux, Sanae Chaib, Nicolas Macagno, Mariame Chraibi, Franck Tirode, Marie Karanian

**Affiliations:** ^1^ Department of Biopathology Centre Leon Berard Lyon France; ^2^ Preclinical Basic Sciences Laboratory, Faculty of Medicine and Pharmacy of Tangiers Abdelmalek Essaadi University Tetouan Morocco; ^3^ Centre de Recherche en Cancérologie de Lyon, INSERM U1052 ‐ CNRS UMR5286, Centre Léon Bérard Université Lyon 1 Lyon France; ^4^ Department of Pathology and Neuropathology La Timone Hospital Marseille France; ^5^ INSERM MMG Aix Marseille University Marseille France

**Keywords:** Chondroid neoplasm, *FOS::PABPN1*, gene fusion, RNA sequencing, soft tissue tumor, synovial chondromatosis

## Abstract

Soft tissue tumors with chondroid matrix represent a heterogeneous group with persistent diagnostic challenges. Advances in molecular diagnostics have identified recurrent gene fusions in several chondroid neoplasms, predominantly involving *FN1.* Here, we report two cases of chondroid tumors harboring a novel *FOS::PABPN1* fusion. The patients were young and presented with small, deep, peri‐osseous nodules located in the extremities. Histologically, the tumors were well‐circumscribed and lobulated. Both cases presented fibro‐cartilaginous and spindle cell areas. No cytonuclear atypia, mitoses, or necrosis were observed. Immunohistochemistry (performed in Case 2) revealed positivity for *S100, CD34,* and *FOS*, with negativity for *MDM2, HMGA2*, *PLAG1*, and desmin. RNA sequencing identified an identical in‐frame *FOS::PABPN1* fusion transcript in both tumors. Unsupervised transcriptomic clustering positioned the cases near synovial chondromatoses and other chondroid lesions, despite the absence of *FN1* rearrangements. Massive overexpression of the *WIF1* (*Wnt* Inhibitory Factor1) gene was also observed. To our knowledge, this is the first description of FOS rearrangement in mesenchymal tumors exhibiting chondroid differentiation. The *FOS::PABPN1* fusion expands the spectrum of *FOS*‐altered neoplasms beyond osteoblastic and vascular lesions and defines a potential new molecular subset of benign chondroid tumors. These findings highlight the utility of RNA sequencing in the diagnosis of chondroid neoplasms in order to identify rearrangement (of *FN1, THBS1* or *FOS*) or *IDH1/2.* The consistent upregulation of *WIF1* may represent a potential diagnostic biomarker.

## 1 Introduction

Mesenchymal tumors are a diagnostic dilemma for pathologists. Advances in histopathology and molecular biology have significantly improved the classification of soft tissue tumors [1, 2, 3, 4]. These advances have led to the emergence of molecular biology as a vital tool in diagnosing different types of soft tissue tumors [2]. These techniques have helped in moving beyond morphological classification of soft tissue tumors and adopting a comprehensive approach wherein molecular biology acts as one of the primary diagnostic hallmarks. Soft tissue tumors with chondroid matrix are a diverse group of neoplasms, and diagnostic difficulties are encountered in precisely determining the type of matrix and its non‐specificity [5]. Advances in molecular biology investigations have helped in discovering new molecular biology subclasses of chondroid mesenchymal neoplasms, such as calcified chondroid mesenchymal neoplasms (CCMN) with FN1 fusions [6]. Recently characterized acral fibrochondromyxoid tumors bearing *THBS1::ADGRF5* fusions and acral chondromyxoid tumor with a *TCF4::ERG* gene fusion have also been described [7, 8, 9]. In addition, OGT‐rearranged mesenchymal neoplasms have recently been identified, further expanding the molecular diversity of soft tissue tumors with myxoid or chondroid features [10].

To the best of our knowledge, no rearrangements involving the *FOS* gene have been reported in tumors displaying chondroid differentiation. The current study documents the discovery of a novel rearrangement, namely the *FOS::PABPN1* fusion, detected in two cases of soft tissue tumors with chondroid matrix—one located in the hand and the other in the foot. This investigation offers fresh insights into the phenotypic diversity of tumors linked to the *FOS* gene family and augments existing literature by proposing a potential role for *FOS* in the pathogenesis of such lesions.

## 2 Materials and Methods

### 2.1 Immunohistochemistry

The following primary antibodies were used for one case: anti‐Desmin (1/80; mouse monoclonal, clone D33; Agilent Technologies DAKO), anti‐ERG (1/1; rabbit monoclonal, clone EPR3864; Roche), anti‐HMGA2 (1/1000; rabbit polyclonal; Biocheck), anti‐PLAG1 (1/100; mouse monoclonal, clone 3B7; Bio‐Techne France), anti‐FOS (1/25; rabbit monoclonal, clone 5G4; Cell Signaling Technology). Immunostaining was performed on a VentanaBenchmark XT automatic stainer (Ventana, Tuscon, AZ) and revealed with the Ultraview Universal Dab Detection kit. Anti‐PS100 (polyclonal; Roche), anti‐CD34 (clone QBEnd/10; Roche), and anti‐MDM2 (clone IF2‐AV; concentrated, Diagomics) antibodies were used externally before sending the case for expert consultation.

### 2.2 RNA Sequencing

Exome‐based RNA capture sequencing was performed on formalin‐fixed paraffin‐embedded (FFPE) samples for both cases, and the data were further analyzed to detect fusion genes and small nucleotide variations (SNVs) and to compare expression profiles with > 10 000 other samples from the CLB molecular database using clustering methods. The molecular basis of the technique, the technical protocol, and the bioinformatics algorithms used have been described previously [11]. Uniform manifold approximation and projection (UMAP) were performed in the R (v4.4.1) environment. Details of 71 control cases used for transcriptomic clustering analyses are provided in Table S1.

## 3 Results

### 3.1 Clinical Findings

Table 1 summarizes the clinical, morphologic, immunohistochemical, and molecular features of the two cases. Both patients were young (30 years for Case 1; 15 years for Case 2) and presented with peri‐osseous soft tissue lesions located in the metatarsal region (Case 1) and the thumb (Case 2). For Case 2, MRI demonstrated a nodule of the right thumb with a deep cystic component; however, the images themselves could not be obtained from the referring institution. No radiological information was available for Case 1. Both patients underwent complete surgical excision. Follow‐up data were available for both patients: Case 1 was followed for 38 months without evidence of local recurrence, and Case 2 was followed for 14 months without recurrence.

**TABLE 1:** Clinical, morphologic, immunohistochemical, and molecular features of the two cases.

Parameter	Case 1	Case 2
Clinical parameters		
Age (years)	30	15
Sex	Female	Male
Size (mm)	20 × 18 × 12	19 × 22 × 11
Location	Foot, metatarsal region (third intermetatarsal space)	Right hand, nodule on thumb (D1)
Depth	Deep	Deep
Relation to bone	Peri‐osseous soft tissue	Peri‐osseous soft tissue
Complete excision	Yes	Yes
Treatment	Surgical	Surgical
Follow‐up (number of months)	38 months (no recurrence).	14 months (no recurrence).
Morphologic and phenotypic parameters		
Gross description	Well‐circumscribed whitish nodular lesion	Solid whitish fragment
Architecture	Well‐circumscribed nodular	Well‐circumscribed nodular
Matrix	Pure chondroid	Mixed fibro‐cartilaginous
Cellular arrangement	Intercrossing bundles + lobules	Intercrossing bundles + lobules
Cell type	Spindle cells and Chondrocytes	Spindle cells and chondrocytes
Nuclei	Ovoid; nucleolated; no binucleation; no cytonuclear atypia	Ovoid; no cytonuclear atypia
Mitoses/necrosis	0/0	0/0
Immunophenotype (IHC)	Not performed	S100+, CD34+, FOS+, MDM2‐, HMGA2‐, PLAG1‐, Desmin—
Molecular data		
Fusion transcript	*FOS::PABPN1*	*FOS::PABPN1*
Transcriptomic clustering	Stably clusters near three lesions: (1) an unclassified tumor with FOS gene rearrangement (initial suspicion of synovial chondromatosis), (2) two lesions labeled as synovial chondromatosis (one with *FN1::ACVR2B* rearrangement)	Clusters with chondroid‐annotated lesions, including one with the same *FOS::PABPN1* fusion transcript (i.e., Case 1); considered less informative overall
Diagnostic conclusion	*FOS*‐rearranged chondroid tumor; unable to definitively classify into a more precise histologic type (expression profile shows similarities to synovial chondromatosis, but *FOS* rearrangement not described in that entity)	Likely benign *FOS*‐rearranged chondroid tumor; unable to definitively classify into a more precise histologic type

### 3.2 Microscopic Findings

Morphologically, these tumors exhibited a well‐circumscribed nodular architecture. They were characterized by a fibrous component at the periphery and a chondroid center. The chondroid matrix consisted of mature chondrocytes organized in small lobules, lacking cytonuclear atypia, binucleation, or mitotic activity. The peripheral fibrous component comprised interlacing bundles of spindle cells without evidence of atypia (Figure S1).

**FIGURE 1:**
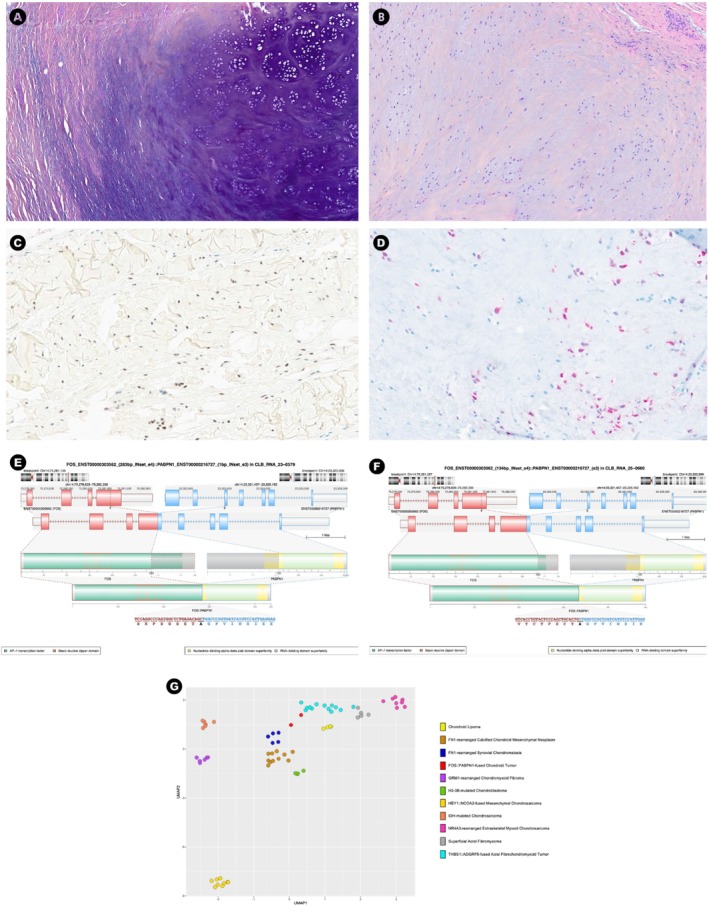
(A) HES stain: Interlacing bundles of spindle cells at the periphery and lobules of cartilage composed of chondrocytes without atypia at center (200×). (B) HES stain: Interlacing fascicles and lobules composed of spindle cells and chondrocytes without atypia (200×). (C) *PS100* immunohistochemistry: Cytoplasmic and nuclear expression in the Tumor cells. (D) *FOSB* immunohistochemistry: Hétérogeneous nuclear expression in the Tumor cells. (E) and (F) Reakpoint characterization of two *FOS::PABPN1* gene fusions by RNA sequencing. (G): Clustering analyses. The clustering analysis by UMAP projection of the *FOS::PABPN1* case, annotated in red, clustered closely with the *THBS1::ADGRF5*‐fused Acral Fibrochondromyxoid Tumor and FN1‐rearranged Synovial Chondromatosis.

This pattern evokes the diagnosis of calcified chondroid mesenchymal neoplasms (CCMN), a recently described category of soft tissue tumors frequently associated with fibronectin 1 (*FN1*) fusions or an acral fibrochondromyxoid tumor (Figure 1A,B).

Immunohistochemical analysis of Case 2 revealed positivity for *S100* and *CD34*, and heterogeneous staining for *FOS*, while lacking expression of *MDM2, HMGA2, PLAG1*, and desmin. The lack of *HMGA2* and *PLAG1* expression was an important finding that differentiates these tumors from soft tissue pleomorphic adenomas that are known for rearrangements of *HMGA2* and *PLAG1*, respectively. Positivity for *CD34* is a rather nonspecific finding for spindle‐cell tumors of mesenchymal origin, but the expression of S100 protein confirms chondroid differentiation within the lobules. *FOS* antibody was tested after the identification of the fusion, and it was expressed by some cells (Figure 1C,D).

The lack of cytological atypia, mitotic activity, and necrosis for both cases is relevant (0/0 for both), indicating that these tumors are likely benign, for which the proposed treatment is conservative surgery.

### 3.3 Molecular Findings

The main molecular finding common to both instances was the presence of the fusion transcript *FOS::PABPN1* (Figure 1E,F). In both *FOS::PABPN1* fusion transcripts, the breakpoint in *FOS* occurs within exon 4. The predicted fusion proteins retain the N‐terminal portion of *FOS*, including the AP‐1 transcription factor domain and the basic leucine zipper (bZIP) domain. On the *PABPN1* side, the breakpoint occurs within exon 3. The predicted chimeric proteins include the C‐terminal portion of *PABPN1* containing the RNA‐binding domain, whereas the N‐terminal nucleotide‐binding alpha–beta pleated domain is not retained. Thus, in both fusion variants, the AP‐1 and bZIP domains of FOS are conserved, as well as the C‐terminal RNA‐binding domain of PABPN1, while the N‐terminal nucleotide‐binding domain of PABPN1 is absent.

Transcriptomic analysis comparing *FOS::PABPN1*‐fused tumors to control samples identified a significant overexpression of *WIF1* (*Wnt Inhibitory Factor 1*). *WIF1* expression levels were markedly increased in fusion‐positive cases, with a 38.7‐fold change (LogRatio 5.28). These results reached high statistical significance after Bonferroni correction for multiple testing (*p* < 0.001). Conversely, *FOS* was found to be not overexpressed (or even underexpressed) in these samples. These findings highlight *WIF1* as a key differentially expressed gene associated with the *FOS::PABPN1* fusion profile. According to clustering analysis, the transcriptomic profiles of these two tumors are similar and closely resemble those of synovial chondromatosis and acral fibrochondromyxoïd tumors (Figure 1G). DNA methylation clustering analysis, while beyond the scope of the present study due to limited FFPE DNA quantity, represents a promising future approach to further define the epigenetic relationship of FOS::PABPN1‐rearranged tumors to other chondroid entities.

The final diagnosis in both cases was a *FOS*‐rearranged chondroid tumor. These two tumors share molecular, morphological, and clinical features supporting the hypothesis that they represent a single, as yet undescribed entity among the group of chondroid tumors.

## 4 Discussion

*FOS* is a proto‐oncogene implicated in tumorigenic processes, encoding a subunit of the activator protein‐1 (AP‐1) transcription factor. Originally identified as the cellular homolog of two viral *FOS* oncogenes that induce osteosarcoma in rats and mice, it is also known as the cellular variant of the *FBJ* murine osteosarcoma viral oncogene homolog (c‐*FOS*) [12]. The AP‐1 transcription factor binds to TPA‐responsive elements (5′‐TGAC/GTCA‐3′) in the promoter and enhancer regions of target genes, thereby regulating a broad array of physiological processes (e.g., osteoblast maturation and adipocyte differentiation) and tumorigenic mechanisms, including cell proliferation, oncogenic transformation, tumor invasion, distant metastasis, angiogenesis, stress responses, organogenesis, immune regulation, and cognitive functions [13]. *FOS* gene rearrangements have been documented in osteoblastomas, osteoid osteomas, epithelioid hemangiomas, pseudomyogenic hemangioendotheliomas, and proliferative myositis/fasciitis [13]. To our knowledge, this represents the first report of *FOS* rearrangement in chondroid mesenchymal neoplasms. The identification of the *FOS::PABPN1* fusion in chondroid tumors thus marks a significant advancement in understanding the genetic diversity of mesenchymal neoplasms. This finding implies that activation of the AP‐1 pathway may drive chondroid differentiation, in addition to osteoid differentiation, depending on cellular context or fusion partners.

Most notably, this fusion involves *PABPN1*, also known as poly(A)‐binding protein nuclear 1, with established roles in post‐transcriptional RNA maturation. *PABPN1* is highly expressed ubiquitously and thus likely to take part in regulating the expression of genes across tissues. Mutations in *PABPN1* have been associated with significant RNA damage. The involvement of *PABPN1* in key nuclear post‐transcriptional functions, such as controlling the length of poly(A) tails and exporting polyadenylated RNA, was suggested in studies on mammalian cells. The protein is required for the progressive and processive polymerization of poly(A) tails at the 3′ end of eukaryotic genes. It keeps the tail short, to about 250 nucleotides in length. More recently, *PABPN1* has been implicated in the selection of poly(A) sites and the regulation of long non‐coding RNAs (lncRNAs) [14].

The *FOS::PABPN1* fusion identified in these benign chondroid tumors likely drives oncogenesis through qualitative functional changes rather than simple quantitative overexpression, as our results indicate that the FOS gene is not overexpressed. This suggests that the clinical phenotype is dictated by the chimeric properties of the fusion protein, which consistently preserves the essential functional domains of both partners: the AP‐1 transcription factor and Basic‐leucine zipper (bZIP) domains of *FOS*, and the RNA‐binding domain superfamily of *PABPN1*. In the absence of *FOS* mRNA elevation, the functional impact may be explained by increased nuclear stability or retention due to *PABPN1*'s nuclear‐specific localization, or by defects in RNA processing such as Alternative Polyadenylation (APA), a mechanism known to promote cell division [15, 16]. Furthermore, the significant upregulation of *WIF1* in fusion‐positive cases suggests that the *FOS::PABPN1* protein effectively reprograms the cellular transcriptome, potentially by directly binding to the *WIF1* promoter via the conserved FOS bZIP domain. This induction of a known Wnt pathway inhibitor highlights a specific regulatory shift that defines the unique biological behavior of these tumors [17]. A clear analogy exists with read‐through *BCL2L2::PABPN1* transcripts identified in glioblastomas and prostate cancers, where the fusion of an anti‐apoptotic gene (*BCL2L2*) with *PABPN1* promotes tumor cell survival. In our chondroid tumor cases, the proliferative signal from FOS is similarly coupled to *PABPN1*, potentially hijacking its regulatory machinery to drive the tumor's specific growth profile [18].

Morphologically, these tumors raise questions about the diagnosis of calcified chondroid tumors, acral fibrochondromyxoid tumor, soft tissue chondroma, and synovial chondromatosis. These two tumors share the same localization as acral fibrochondromyxoid tumors and CCMN affecting the hand and feet [7, 19]. Microscopically, these entities are very close to the entity we reported here, but CCMN shows “grungy” or lace‐like calcifications, which were absent in our cases. However, the absence of classical rearrangement identified in these different entities and the presence of FOS fusion necessitate considering these lesions as biologically distinct from these chondroid tumors. CCMN is characterized by *FN1::FGFR1/2* or other kinase partner fusions, and acral fibrochondromyxoid tumor by the fusion *THBS1::ADGRF5*. Soft tissue chondroma (STC), typically benign and well‐circumscribed, has been associated with *FN1::FGFR1/2* fusions in some instances [20]. The differential diagnosis with STC is warranted given the shared acral location and chondroid differentiation. However, STC typically lacks a peripheral spindle cell fibrous component and is characterized by *FN1* gene rearrangements, whereas our cases display a biphasic histology and a novel *FOS::PABPN1* fusion without *FN1* alterations. Therefore, we propose that these tumors are best considered as a potential new entity distinct from STC, provisionally termed ‘*FOS*‐rearranged chondroid tumor.’

Clinically, these two tumors differ from synovial chondromatosis, which primarily involves large joints such as the knee, as well as the temporomandibular and intervertebral joints. However, some cases occur extra‐articularly; these are termed tenosynovial chondromatosis and are typically located in the hands and feet. Morphologically, these tumors can be distinguished from synovial chondromatosis by the presence of a peripheral rim of residual synovial tissue surrounding the chondroid proliferation. Synovial chondromatosis is characterized by the presence of fusions involving the *FN1* gene [20, 21]. The presence of *FOS* fusion in this morphologic context represents a novel finding [22].

The heterogeneous immunohistochemical expression of *FOS*, occurring despite the absence of *FOS* gene overexpression at the transcript level, is likely attributable to a post‐translational stabilization mechanism inherent to the fusion structure. Recurring rearrangements involving the fourth exon of the *FOS* gene lead to truncation at the C‐terminus, eliminating the “LLAL” (Leu‐Leu‐Ala‐Leu) sequence. Physiologically, this sequence targets the protein for ubiquitin‐independent proteasomal degradation, limiting the duration of the wild‐type *FOS* protein expression. However, the degradation mechanism is circumvented in the *FOS::PABPN1* fusion protein, thus rendering the protein stable at levels detectable via immunohistochemistry, irrespective of the baseline mRNA levels [23].

Furthermore, the high‐level transcriptional upregulation of the *WIF1* gene indicates that anti‐*WIF1* immunohistochemistry can be used as an extremely sensitive diagnostic tool for this tumor. *WIF1* is a secreted antagonist of the *Wnt* signaling pathway and plays an essential role in mesenchymal and chondroid differentiation. Given the significant induction of *WIF1* observed in *FOS::PABPN1*‐rearranged cases (exhibiting a LogRatio of 5.28 in our series), this marker provides a sensitive tool for the identification of these lesions in routine practice. Integrating *WIF1* into the immunohistochemical panel may prove essential for distinguishing this novel entity from morphological mimics like synovial chondromatosis or CCMN, which do not typically exhibit such a profound and specific *WIF1* expression profile [24, 25].

Regarding the biological behavior of these lesions, the absence of cytonuclear atypia, mitotic activity, and necrosis strongly supports a benign clinical course. This is further supported by the available follow‐up data (38 months for Case 1 and 14 months for Case 2, both without recurrence). However, given the novelty of this *FOS::PABPN1* fusion and the limited number of cases described to date, further studies with long‐term follow‐up are essential to definitively characterize their clinical behavior and confirm their place within the spectrum of benign chondroid neoplasms. We acknowledge that complete clinical data could not be obtained for these consultation cases. MRI images were not available for Case 1, and while Case 2 had MRI showing a nodule with a deep cystic component, the images themselves could not be obtained. Despite these limitations, the available clinical and pathological data support the benign nature of these lesions.

## 5 Conclusions

Our study identifies a potential new biological entity within chondroid soft tissue tumors, defined by the recurrent *FOS::PABPN1* fusion. This discovery extends the oncogenic role of the *FOS* gene family beyond the classically known osteoblastic and vascular lesions, suggesting that activation of the AP‐1 pathway may also induce chondroid differentiation depending on the cellular context. Diagnostically, these tumors share morphological similarities with synovial chondromatosis and CCMN, but they are distinguished by their unique molecular signature and the increased fusion protein stability. The upregulation of *WIF1* observed in our cases provides a promising diagnostic for routine practice. However, as this is an initial description, further studies are needed to understand the relationship between this entity and other chondroid tumors and to clarify its long‐term clinical behavior. The systematic integration of RNA sequencing within expert networks will remain essential for documenting new cases and confirming the benign nature of this molecular subgroup.

## Funding

The authors have nothing to report.

## Ethics Statement

The authors have nothing to report.

## Conflicts of Interest

The authors declare no conflicts of interest.

## Supporting information


**Figure S1:** Histological features of *FOS::PABPN1*‐rearranged chondroid tumors (H&E staining). The peripheral fibrous component showing bland spindle cells arranged in loose fascicles, lacking cytological atypia or mitotic activity (A: 200×, B: 400×). Note the presence of cleared, vacuolar spaces within the stroma in (B); these represent degenerative/stromal vacuolar changes rather than true adipocytes or an infiltrative margin.

**Table S1:** Quality control metrics, clinical metadata, and molecular profiles of the 71control samples utilized for transcriptomic clustering analysis. Technical sequencing parameters, patient demographics, anatomical sites, histopathological diagnoses, mutational/fusion status, and pathway signature scores (CINSARC, G2M) are reported for each control case.

## Data Availability

The data that support the findings of this study are available from the corresponding author upon reasonable request.
