# Supplementary material for: Chondroid Soft Tissue Tumors With FOS::PABPN1 Fusion: A New Entity? About Two Cases
Source: Genes Chromosomes Cancer. 2026 Aug 7;65(8):e70157. doi: 10.1002/gcc.70157 (PMC13449868; doi:10.1002/gcc.70157)
Supplement: Supplementary file 1 [file GCC-65-e70157-s002.docx]

Figure 1S: Histological features of *FOS::PABPN1*-rearranged chondroid tumors (H&E staining). The peripheral fibrous component showing bland spindle cells arranged in loose fascicles, lacking cytological atypia or mitotic activity (A: 200×, B: 400×). Note the presence of cleared, vacuolar spaces within the stroma in (B); these represent degenerative/stromal vacuolar changes rather than true adipocytes or an infiltrative margin.
